# Lithium-Associated Thyromegaly: An Unusual Cause of Airway Obstruction

**DOI:** 10.1155/2012/627415

**Published:** 2012-09-09

**Authors:** Ashish Verma, Siddharth Wartak, Mark Tidswell

**Affiliations:** Baystate Medical Center, Tufts University School of Medicine, Springfield, MA 01199, USA

## Abstract

Acute upper airway obstruction is a medical emergency and can be caused by many serious conditions such as a foreign body occluding the airway, intrinsic swelling (as in anaphylaxis), or extrinsic compression. Thyromegaly has rarely been reported as a source of airway compromise. We present a patient whose thyromegaly is presumed to have been induced by lithium and was massive enough to cause severe airway compromise.

## 1. Introduction

Lithium has been used for treatment of bipolar disorder and depression for over six decades [1]. Multiple studies have shown that thyroid disorders are common in patients undergoing lithium therapy. Subclinical thyroiditis is common, followed by goiter and hypothyroidism. Very rarely, lithium can induce thyromegaly and thyrotoxicosis [2]. We present a case of severe upper airway obstruction and respiratory failure from massive thyromegaly due to lithium associated thyrotoxicosis.

## 2. Case Presentation

In February 2007, a 42-year-old man was brought to our hospital with seizures and respiratory distress. He had progressive shortness of breath and fatigue for a few days before this presentation. His past medical history and treatment were primarily at another institution and were significant for mental retardation, seizure, disorder and an asymptomatic cerebral arteriovenous malformation. In 1995, the patient's psychiatrist commenced him on lithium for treating his cognitive impairment and labile mood. In 2001, he was diagnosed with a thyrotoxic goiter and was prescribed Methimazole. Records of thyroid function from this time were unavailable. A year later, he underwent a lengthy surgical procedure at another hospital that was only partially successful in that the right lobe of his thyroid was resected, with the left lobe and substernal portion left behind. Following this surgery, he made significant recovery. He was followed by an otolaryngologist as outpatient and did not have any significant upper airway compression to necessitate any further resection of the remaining goiter tissue. Patient remained on lithium and Methimazole until this presentation in 2007. His outpatient medications at presentation included Prevacid 30 mg qd, Percocet prn, Methimazole 12.5 mg qd, lithium 600 mg bid, Seroquel 25 mg am and 50 mg qhs, lorazepam 1 mg bid, Dilantin 200 mg bid, Mirapex 0.125 mg tid, Zoloft 100 mg bid, and Colace prn.

## 3. Management

On arrival to hospital, he was in severe respiratory distress. Attempts for an oral endotracheal intubation were unsuccessful because of extreme right-sided tracheal deviation secondary to the left neck mass. Patient underwent an emergent tracheotomy in the emergency room, was stabilized on mechanical ventilator, and transferred to the critical care unit.

**Figure 1:**
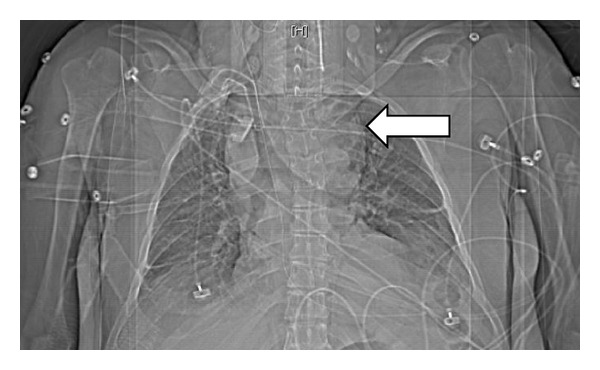
Massive retrosternal thyroid.

**Figure 2:**
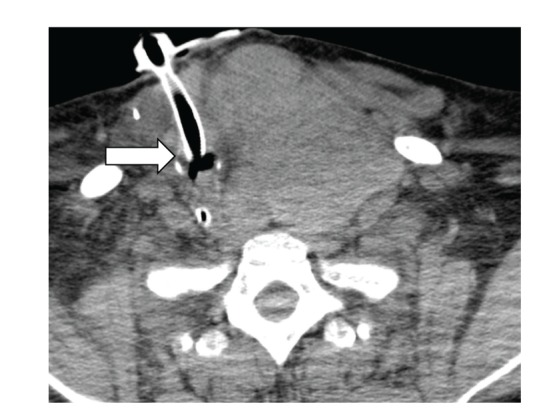
Tracheal deviation and narrowing (transverse view). Also seen is the tracheostomy tube.

**Figure 3:**
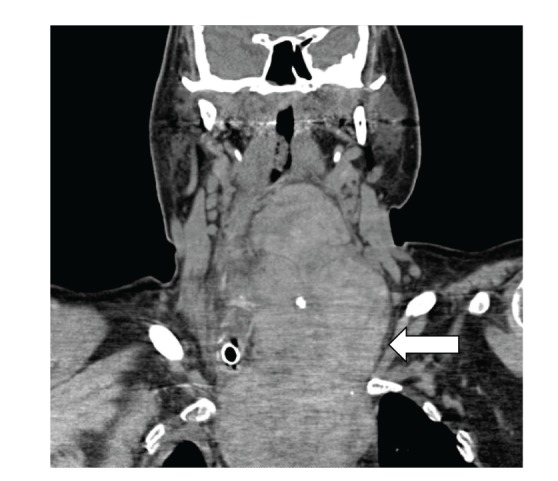
Large cervicomediastinal mass (coronal view).

**Figure 4:**
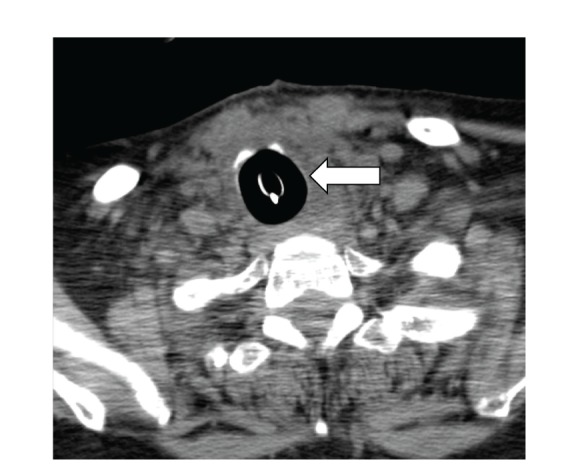
Postthyroidectomy airway.

A CT scan done (Figures 1, 2, and 3) showed massive thyromegaly extending from base of tongue to just below the aortic arch. The thyroid gland measured 10 × 8 cm at the thoracic inlet. It caused deviation and significant narrowing of the trachea in the superior mediastinum and above the carina. The patient was gradually weaned from mechanical ventilator support. The common causes of thyrotoxicosis and thyromegaly were ruled out. The timeline of events points to lithium as the culprit drug for inducing this massive thyromegaly. Lithium was withdrawn and a successful thyroidectomy of the remaining tissue was performed at a subsequent admission. A CT scan performed after the surgery showed a dramatic improvement in the entire airway dimensions with no residual mass (Figure 4).

## 4. Discussion

Thyroid abnormalities due to lithium have protean manifestations. Silent thyroiditis is the most common, followed by goiter and hypothyroidism. Amongst patients on lithium therapy, goiter and hypothyroidism occur in 50% and 20%, respectively [3]. Toxic nodular goiter and Grave's hyperthyroidism occur less frequently. Lithium-associated thyrotoxicosis is unusual with an estimated incidence of 2.7 per thousand patient years treated with lithium [2]. The incidence of thyrotoxicosis was 2-3-fold higher in lithium-treated patients compared to the general population [4]. Hypo- or hyperthyroidism is more common in women than in men and incidence increases with age [5].

Lithium may induce goiter and hypothyroidism by inhibiting formation and secretion of T3 and T4. This is due to lithium-induced increment in the serum thyroid antibody concentrations [6]. In animal models, it has been shown that lithium may influence DNA synthesis of thyroid cell to stimulate goiter formation [7]. About 10–33% of patients on long-term lithium therapy may have high serum thyroid antibody levels [8]. Mechanism of thyroid toxicity may be that lithium is concentrated in the thyroid gland and damages follicular cells, causing release of thyroglobulin into the circulation. Alternatively, lithium may induce a painless sporadic thyroiditis [4, 9].

Thyroid gland enlarges gradually over years, either diffusely or with nodules. It has a tendency to enlarge downwards into the superior mediastinum as it is bound by rigid structures and this is the path of least resistance. Furthermore, there is downward traction due to negative intrathoracic pressure, swallowing, and weight of the gland itself due to lack of any fascia plane inferiorly [10]. This imposes a compression effect into the superior mediastinal structures, mainly the upper airway. A goiter compressing the upper airway is rare, but when seen is more common for substernal goiter. Depending on the population studied, the risk is 16–85% [11]. The consensus for effective treatment for substernal goiter is surgery due to the high risk of airway compression and low risk of the surgery [10, 12]. 

Our patient had a rare manifestation of lithium causing thyrotoxicosis and massive thyromegaly. This progressed to a point causing upper airway obstruction and asphyxia requiring emergent tracheostomy and urgent thyroidectomy. The enlargement was over years and the initial attempt for a complete surgical resection had failed due to intraoperative complication. The patient was followed closely as outpatient, and was doing well, but unfortunately his thyromegaly progressed to cause acute airway compression. The patient received definite management by surgical resection of the remaining enlarged goiter tissue. This case adds to the literature the knowledge of lithium's almost fatal side effect. As a clinician, it is very important to perform regular lithium assay and regular thyroid monitoring. Finally, the substernal goiters are best managed surgically. Treatment is the same as for other forms of thyrotoxicosis and withdrawal of the offending drug is advisable.

## 5. Conclusion

Lithium-associated thyrotoxicosis and thyromegaly are rare but important adverse effects of lithium therapy, which, if regularly monitored, may reduce complication in this patient group. Surgery is an effective and preferred treatment for thyroid enlargements causing airway compression, especially for substernal goiter.
